# Identification of *Microcystis aeruginosa* Peptides Responsible for Allergic Sensitization and Characterization of Functional Interactions between Cyanobacterial Toxins and Immunogenic Peptides

**DOI:** 10.1289/ehp.1409065

**Published:** 2015-04-22

**Authors:** Esmond N. Geh, Debajyoti Ghosh, Melanie McKell, Armah A. de la Cruz, Gerard Stelma, Jonathan A. Bernstein

**Affiliations:** 1 Allergy Section, Division of Immunology Allergy, and Rheumatology, Department of Internal Medicine, and; 2 Department of Environmental Health, University of Cincinnati College of Medicine, Cincinnati, Ohio, USA; 3 Miami University, Oxford, Ohio, USA; 4 Office of Research and Development, U.S. Environmental Protection Agency, Cincinnati, Ohio, USA

## Abstract

**Background:**

The cyanobacterium species *Microcystis aeruginosa* produces microcystin and an array of diverse metabolites believed responsible for their toxicity and/or immunogenicity. Previously, chronic rhinitis patients were demonstrated to elicit a specific IgE response to nontoxic strains of *M. aeruginosa* by skin-prick testing, indicating that cyanobacteria allergenicity resides in a non-toxin–producing component of the organism.

**Objectives:**

We sought to identify and characterize *M. aeruginosa* peptide(s) responsible for allergic sensitization in susceptible individuals, and we investigated the functional interactions between cyanobacterial toxins and their coexpressed immunogenic peptides.

**Methods:**

Sera from patients and extracts from *M. aeruginosa* toxic [MC(+)] and nontoxic [MC(–)] strains were used to test IgE-specific reactivity by direct and indirect ELISAs; 2D gel electrophoresis, followed by immunoblots and mass spectrometry (MS), was performed to identify the relevant sensitizing peptides. Cytotoxicity and mediator release assays were performed using the MC(+) and MC(–) lysates.

**Results:**

We found specific IgE to be increased more in response to the MC(–) strain than the MC(+) strain. This response was inhibited by preincubation of MC(–) lysate with increasing concentrations of microcystin. MS revealed that phycocyanin and the core-membrane linker peptide are the responsible allergens, and MC(–) extracts containing these proteins induced β-hexosaminidase release in rat basophil leukemia cells.

**Conclusions:**

Phycobiliprotein complexes in *M. aeruginosa* have been identified as the relevant sensitizing proteins. Our finding that allergenicity is inhibited in a dose-dependent manner by microcystin toxin suggests that further investigation is warranted to understand the interplay between immunogenicity and toxicity of cyanobacteria under diverse environmental conditions.

**Citation:**

Geh EN, Ghosh D, McKell M, de la Cruz AA, Stelma G, Bernstein JA. 2015. Identification of *Microcystis aeruginosa* peptides responsible for allergic sensitization and characterization of functional interactions between cyanobacterial toxins and immunogenic peptides. Environ Health Perspect 123:1159–1166; http://dx.doi.org/10.1289/ehp.1409065

## Introduction

Cyanobacteria (formerly known as blue-green algae) are ubiquitous photosynthetic bacteria that have the potential to produce toxins. Cyanobacteria are primarily found in freshwater systems worldwide. In nutrient-rich water, cyanobacteria cells proliferate to form a mass called a bloom. During the past decade, cyanobacteria blooms have been of increasing concern to public health and water management officials as their potential health effects are being better recognized. Global climate change, resulting in increases in water temperatures and severe droughts in combination with increases in nutrient load, has led to massive and prolonged cyanobacteria blooms in many large bodies of freshwater in the United States, further threatening human health and the environment (O’Neil et al. 2012). Specifically, individuals living in close proximity to these bodies of water and/or those who use them for recreational activities are at risk for increased exposure to cyanobacteria. However, recent reports have found cyanobacteria species in homes remote from outdoor water sources (Konya et al. 2014). Exposure to cyanobacteria is primarily from accidental ingestion of contaminated water while engaging in recreational activities or consuming food supplements containing cyanobacteria (Gilroy et al. 2000; Rellán et al. 2009; Vichi et al. 2012). In addition, exposure can also occur through direct skin contact (Codd et al. 1999) with contaminated water or by inhalation when cyanobacteria become aerosolized (Wood and Dietrich 2011). Because the number of reported cyanobacteria blooms appears to be increasing each year, there is greater risk of human exposure to these organisms.

Significant variability exists in the toxicity of cyanobacteria because some species produce toxins but others do not (Saker et al. 2005). Interestingly, animal studies have shown adverse health effects despite the lack of measurable known cyanotoxins (Bernard et al. 2003; Fastner et al. 2003; Griffiths and Saker 2003; Saker et al. 2003); this suggests that cyanobacteria blooms can lead to different health-effect outcomes, depending on whether the bloom is toxic or nontoxic. For example, cyanobacteria have been demonstrated to sensitize susceptible individuals who are reported to develop itchy rashes and eye irritation, or other hay fever–like upper respiratory symptoms, after swimming in contaminated water (Pilotto et al. 1997). These symptoms could reflect the direct toxic effect or an allergic reaction to a toxin and/or coexpressed allergenic peptide.

A number of clinical studies in humans found a significant correlation between exposure to cyanotoxins and allergic reactions in sensitized individuals (Mittal et al. 1979; Pilotto et al. 1997; Stewart et al. 2006a, 2006b). Using non-toxin–producing strains of cyanobacteria (*Microcystis aeruginosa, Arthrospira platensis,* and *Aphanizomenon-flos aquae*), Bernstein et al. (2011) found that 74 (28%) of 259 chronic rhinitis patients examined were positive for cyanobacteria by skin-prick testing (SPT). The conclusion from that study was that cyanobacteria allergenicity likely resides in a non-toxin–producing component of the organism. Therefore, the purpose of the present study was to identify and characterize the cyanobacteria allergen(s) responsible for causing sensitization in these individuals and to better understand the relationship between cyanobacteria allergenicity and toxicity.

## Materials and Methods

*Reagents and antibodies*. Goat anti-human IgE, alkaline phosphatase–conjugated rabbit anti-goat IgG, and horseradish peroxidase (HRP)-conjugated rabbit anti-goat IgG were purchased from KPL (Gaithersburg, MD). We obtained anti-phycocyanin from Bioss (Woburn, MA), microtiter ELISA plates from Corning (Corning, NY), microcystin LR and anti-FcεRI from EMD Millipore Corporation (Billerica, MA), Eagle’s Minimum Essential Medium (EMEM) from ATCC (Manassas, VA), and phycocyanin, G418, and pNPP substrate from Sigma-Aldrich (St. Louis, MO).

*Study population*. After signing an Institutional Review Board–approved informed consent and, if applicable, an assent, participants ≥ 6 years of age who were being evaluated for allergic rhinitis with aeroallergen skin testing also underwent SPT to extracts from *M. aeruginosa*–nontoxic [MC(–)] strains. Due to ethical concerns, SPT to extracts from *M. aeruginosa*–toxic [MC(+)] strains was not performed. Participants with dermatographia or who were taking medications that prohibited skin testing were excluded from the study. A chart review was conducted for each participant to obtain information regarding potential sources of indoor cyanobacteria exposure. All allergic and control participants were recruited locally and living in the greater Cincinnati, Ohio, area.

*Serum*. The patient sera used for this study were collected from a previous study that longitudinally enrolled and skin tested 259 patients presenting with chronic rhinitis over 2 years (Bernstein et al 2011). Serum was collected from a subset of 15 patients who elicited strong skin test responses and from 3 nonatopic healthy control subjects. All serum was aliquoted and stored at –20°C until use. To conserve serum, after performing an initial IgE-specific ELISA with the individual serum, sera from 4 of the patients showing strong IgE reactivity were pooled using equal volumes and used in all subsequent experiments. For controls, sera from 3 nonatopic healthy subjects were pooled and used in every experiment.

*Cultivation of* M. aeruginosa *and preparation of crude cell extracts*. Axenic cultures of *M. aeruginosa* (2385 and 2386) were obtained from UTEX The Culture Collection of Algae (University of Texas at Austin, Austin, TX). *M. aeruginosa* 2385 produces the cyanobacterial toxin microcystin [toxic, MC(+)], whereas 2386 does not produce microcystin toxin [nontoxic, MC(–)]. Cultures were cultivated in BG11 broth medium (Sigma-Aldrich, St. Louis, MO) supplemented with 1.8 mM sodium nitrate NaNO_3_ and 10 mM sodium bicarbonate. Cultures were incubated and maintained under fluorescent white light (irradiance incident of 20 μmol/m^2^/sec) at 25°C without mixing, with a 14:10 hr light and dark cycle and humidity at 55%. Cells in logarithmic phase (absorbance at 600 nm, ~ 0.7) were harvested by centrifugation (3,200 × *g*, 15 min, 4°C) and washed three times with sterile 0.01 M phosphate-buffered saline (PBS), pH 7.4. Washed cells were kept at –20°C until needed.

To obtain the crude extract, 50 mg of washed *M. aeruginosa* cells were resuspended in 1 mL lysis buffer (10 mM Tris-HCl, pH 8.0, 1 mM EDTA). The resuspended cells were then incubated on ice for 20 min. The cells were then sonicated using a cell disruptor (Heat System, Ultrasonics Inc., New York, NY) at setting 7 three times for 20 sec, followed by 40-sec incubation on ice. The extract was centrifuged at 14,000 × *g* for 30 min at 4°C. The supernatant was then collected and either lyophilized or concentrated using Amicon Ultra-15 (10 kDa capacity) centrifuge tubes (EMD Millipore Corporation) before freezing at –80°C. The total protein yield for both the MC(–) and MC(+) was approximately 1.2 mg per 50 mg dry cell weight. The concentrated lysates were used in all experiments except the toxicity studies. For the toxicity assay, we used lyophilized lysates because the Amicon tubes were inefficient in retaining low-molecular-weight proteins such as microcystin (approximately 1,000 Da).

*IgE-specific ELISA*. IgE-specific ELISA was performed using a previously described method (Davies et al. 2011). Briefly, 96-well polystyrene microtiter plates were coated with 100 μL/well of 10 μg/mL lysates in 50 mM carbonate buffer, pH 9.6. The plates were incubated for 2 hr at room temperature (RT) and then overnight at 4°C. After overnight incubation, the wells were then emptied, washed four times for 5 min each with 0.05% Tween-20 in 0.01 M PBS, pH 7.4 (PBS-T), and blocked for 1 hr with blocking buffer [2% bovine serum albumin (BSA) in PBS-T]. Serum samples from patients and controls (100 μL from each, diluted 1:10 with blocking buffer) were added to the wells and further incubated for 1 hr at RT and then overnight at 4°C. The wells were washed four times with blocking buffer and incubated with goat anti-human IgE (diluted 1:1,000 with blocking buffer) for 2 hr at RT, then washed again and incubated with alkaline phosphatase–conjugated rabbit anti-goat IgG (1:1,500). The plates were developed using *p*-nitrophenyl phosphate (pNPP) substrate and read at 405 nm using a Multiskan Ascent 96 Plate Reader (Thermo Scientific, Madison, WI). The results are presented as optical density (OD) or absorbance compared with controls (relative OD).

*ELISA inhibition*. Antigen specificity and cross-reactivity were determined using an ELISA competitive inhibition assay. Pooled serum from patients sensitized to *M. aeruginosa* was preabsorbed at 4°C overnight, with increasing concentrations of MC(–) lysate (solution phase). The MC(–) lysate–serum mixture was then added to wells coated with MC(–) and MC(+) lysates (solid phase). Subsequent steps of the inhibition assay were performed as described for the IgE-specific ELISA above.

*SDS-PAGE, immunoprecipitation, and immunoblot analyses*. SDS-PAGE (4% stacking, 12% resolving) of *M. aeruginosa* lysates was carried out following the method of Laemmli (1970); 5 μg protein was loaded per lane and electrophoresed in reduced conditions. Resolved protein bands were transferred onto polyvinylidene fluoride membranes, blocked with 5% nonfat milk, and incubated with 1:10 dilution of SPT-positive patient’s serum in blocking buffer (2% BSA in PBS-T). Protein bands recognized by the patient’s IgE were detected using goat anti-human IgE followed by HRP-conjugated rabbit anti-goat IgG. The membrane was incubated with LumiGLO Chemiluminescent substrate (KPL) for 10 min, followed by exposure using a ChemiDoc apparatus (Ultra-Violet Products Ltd, Upland, CA).

For immunoprecipitation (IP), lysates were precleared by incubating with 10 μL of Protein A beads in IP Lysis Buffer (Pierce, Rockford, IL). The precleared lysate was then preincubated with anti-phycocyanin antibody (1:1,000 dilution) for 1 hr at RT before adding the cyanobacteria lysate. The mixture was further incubated for an additional 1 hr at RT and then overnight at 4°C. The beads were then washed four times with IP wash buffer (Pierce, Rockford, IL). After the last wash, the immune complexes were then eluted using 50 μL of Elution Buffer (Pierce). SDS-PAGE and immunoblot analysis were performed on the eluate as described above.

*Two-dimensional (2D) gel electrophoresis*. 2D gel electrophoresis was performed using 500 μg of cyanobacteria lysate. The lysate was solubilized in 125 μL DeStreak Rehydration Solution (GE Healthcare, Piscataway, NJ) prior to applying to pH 3–11 range strips [Immobiline DryStrip Gels (IPG strips); GE Healthcare, Piscataway, NJ] for isoelectric focusing (IEF). IEF of the proteins was performed on an Ettan IPGphor apparatus (GE Healthcare) using the following running conditions: 500 V for 1 hr, 2,000 V for 1 hr, and 8,000 V for 2 hr. After focusing, the strips were then incubated in equilibration buffer [6 M urea, 75 Mm Tris–HCl, pH 8.8, 30% (vol/vol) glycerol, 2% SDS, 1% DTT, 1% iodoacetamide] for 30 min before being mounted on a 12.5% polyacrylamide gel. Electrophoresis and immunoblotting were carried out as described above. Coumassie staining was performed using Bio-Safe Coomassie Stain (Bio-Rad Laboratories, Hercules, CA) per the manufacturer’s protocol.

*Mass spectrometry*. Nano-liquid chromatography–coupled electrospray tandem mass spectrometry (nLC-ESI-MS/MS). nLC-ESI-MS/MS analyses were performed on a TripleTOF 5600+ (ABSciex, Toronto, Ontario, Canada) attached to an Eksigent (Dublin, CA) nanoLC.ultra nanoflow system. The recovered peptides were loaded (via an Eksigent nanoLC.as-2 autosampler) onto an IntegraFrit Trap Column (outer diameter, 360 μm; inner diameter, 100 μm, and 25 μm packed bed) from New Objective Inc. (Woburn, MA) at 2 μL/min in formic acid/H_2_O, 0.1/99.9 (vol/vol) for 15 min to desalt and concentrate the samples. For the chromatographic separation of peptides, the trap column was switched to align with the analytical column, an Acclaim PepMap100 (inner diameter, 75 μm; length, 15 cm; C18 particle size, 3 μm; pore size, 100 Å) from Dionex-Thermo Fisher Scientific (Sunnyvale, CA). The peptides were eluted using a variable mobile phase gradient from 95% phase A (formic acid/H_2_O, 0.1/99.9, vol/vol) to 35% phase B (formic acid/acetonitrile, 0.1/99.9, vol/vol) for 15 min, from 35% phase B to 80% phase B for 1 min, and then kept at the same mobile phase composition for 2 more minutes at 300 nL/min. The nLC effluent was ionized and sprayed into the mass spectrometer using NANOSpray® III Source (AB Sciex). Ion source gas 1 (GS1), ion source gas 2 (GS2) and curtain gas (CUR), respectively, were kept at 7, 0, and 25 vendor-specified arbitrary units. Interface heater temperature and ion spray voltage was kept at 150°C and 2.5 kV, respectively. The mass spectrometer was operated in positive ion mode set to go through 2,091 cycles for 25 min, with each cycle performing one time-of-flight (TOF)-MS scan type (0.25 sec accumulation time, in a 350–1,500 *m/z* window) followed by 30 information-dependent acquisition (IDA)-mode MS/MS-scans on the most intense candidate ions, having a minimum 250 counts. Each product ion scan was operated under vender-specified high-sensitivity mode with an accumulation time of 0.05 sec and a mass tolerance of 50 mDa. Former MS/MS-analyzed candidate ions were excluded for 15 sec after their first occurrence, and data were recorded using Analyst®-TF (version 1.6; SCIEX, Framingham, MA) software.

Data analyses. Searches from the nLC-MS/MS analyses were accomplished using Mascot Daemon software (version 2.2.2; Matrix Science, Boston, MA), against an NCBInr 20120123 database (http://www.matrixscience.com/) of all entries’ protein sequences. Deamidation of asparagine and glutamine and oxidation of methionine were selected for the search parameters as variable modification, and carbamidomethyl modification of cysteine was used as a fixed modification. The enzyme selected was trypsin, with a maximum missed cleavage of 2. The peptide mass tolerance and the fragment mass tolerance were selected as ± 0.1 Da and ± 0.3 Da, respectively.

*Hexosaminidase release assay*. A β-hexosaminidase release assay, a surrogate assay for measuring histamine release, was used to identify functional activity of *M. aeruginosa* extracts. Rat basophil leukemia cells (RBL SX-38; kindly provided by J.P. Kinet, Harvard Medical School) cultured in complete medium (EMEM supplemented with 10% fetal bovine serum) were seeded onto 96-well plates. One day after reaching confluence, the cells were sensitized with pooled serum from either controls or patients (1:5 dilution in cell culture medium) for 16 hr at 37°C. After sensitization, cells were washed twice with Tyrod’s buffer (10 mL 1 M HEPES, 7.54 g sodium chloride, 0.37 g potassium chloride, 0.206 g calcium chloride, 0.203 g magnesium chloride, 1.008 g glucose, 1 g BSA) and then exposed for 30 min to 100 μg/mL of cyanobacterial lysate and a 1:1,000 dilution of high-affinity IgE receptor (FcεR1) in Tyrod’s buffer. Aliquots of the supernatant (40 μL) were transferred onto 96-well plates and incubated with 40 μL pNAG substrate (1 mM *p*-nitrophenyl-*N*-acetyl-β-d-glucosaminide in 0.05 M citrate, pH 4.5) for 1 hr at 37°C. Total release was determined by lysing the cells with 1% Triton X-100. The reaction was stopped by adding 150 μL of 0.2 M glycine, pH 10.7. The absorbance was measured with a microplate reader (Thermo Scientific) at 405 nm, and the percentage of total β-hexosaminidase was calculated as follows: Percent degranulation = OD supernatant ÷ (OD supernatant + OD Triton X-100) × 100.

*Measurement of microcystin*. Total microcystin was measured using a QuantiPlate™ Kit for Microcystins (EnviroLogix Inc., Portland, ME). All incubations were performed using a shaker incubator (200 rpm; Innova 4080; New Brunswick Scientific, Edison, NJ) at 25°C. Briefly, strain MC(+) lysates were first added to allow binding to anti-microcystin antibodies on the wells. After incubation, microcystin-enzyme conjugate was added, reincubated, and washed and then the substrate added. The enzymatic reaction was stopped, incubated for 10 min and air bubbles eliminated, and then read at 450 nm (650 nm for turbidity blank control) using a Spectra-max M2 spectrophotometer (Molecular Devices, Sunnyvale, CA). All samples were analyzed in triplicate except the kit negative control and the microcystin-LR standard in Advantage A10 Milli-Q water (MQ-H_2_O; Millipore Corp.) in six replicates.

*Cytotoxicity assay*. RBL SX-38 were seeded at 10^4^ cells/well in a 96-well plate. At 90% confluence, the cells were either left untreated or treated for 48 hr with varying concentrations of *M. aeruginosa* toxic [MC(+)] and nontoxic [MC(–)] strain lysates. At the end of the treatment, a CytoScan-WST-1 cell toxicity kit (G-Biosciences, St. Louis, MO) was used to measure the cytotoxic effect of the lysates according to the manufacturer’s protocol. Percent cytotoxicity was calculated as follows: Percent cytotoxicity = [100 × (cell control – experimental)] ÷ (cell control).

*Statistical analysis*. All data are reported as the sample mean ± SD. Comparisons between the means of sample groups and controls were performed using an unpaired Student’s *t*-test. A paired Student’s *t*-test was used to compare IgE reactivity of individual patient serum to MC(–) and MC(+) lysates. Differences were considered statistically significant if the Student *t*-test provided *p*-value < 0.05.

## Results

**Figure 1:**
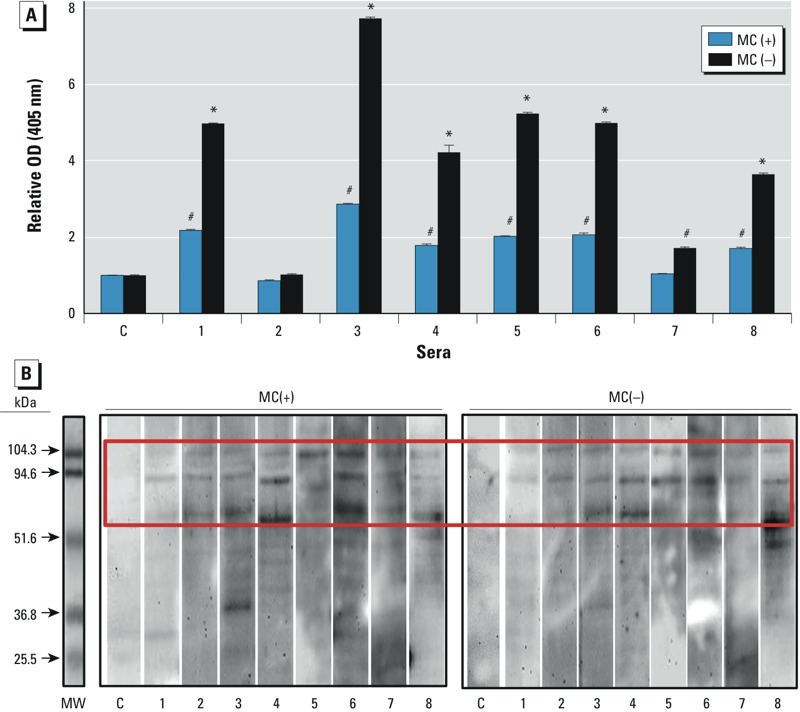
IgE-specific ELISA (*A*) and Western blot analysis (*B*) of individual serum samples from *M. aeruginosa* SPT-positive patients (1–8) and a nonatopic control (C). *M. aeruginosa* crude cell extracts from toxic [MC(+)] and nontoxic [MC(–)] strains were used to detect IgE-specific reactivity to proteins. Aliquots of the extracts used for the ELISA was used to perform the Western blot. MW, molecular weight marker. ELISA results are representative of three separate experiments performed using three different batches of MC(–) and MC(+) lysates (see Supplemental Material, Figure S1). *p < 0.0001, and ^#^*p *< 0.001 for individual patient’s serum compared with pooled control serum, by unpaired Student’s *t*-test.

*Immunoreactivity of* M. aeruginosa *extracts with patient serum*. Table 1 summarizes the demographic characteristics of eight patients who had previously been identified to exhibit a specific IgE response to the cyanobacteria *M. aeruginosa* by SPT (Bernstein et al. 2011). An IgE-specific ELISA was performed using lysates of toxic [MC(+)] and nontoxic [MC(–)] *M. aeruginosa* strains and the sera previously collected from *M. aeruginosa* SPT-positive patients. ELISA results (Figure 1A) demonstrate that sera from these patients differed in their immune reactivity to cyanobacteria peptides. Sera from patients 1, 3–6, and 8 showed strong immunoreactivity with proteins from both lysates. Although the serum from patient 7 reacted significantly (*p* < 0.001) with the *M. aeruginosa* MC(–) lysate, it exhibited no reactivity with the MC(+) lysate. Patient 2 serum had no reactivity with either lysate. Although both strains had a similar immune-reactive profile, the MC(–) lysate was significantly (*p* < 0.01) more immunoreactive than the MC(+) lysate, indicating that the nontoxic strain was more allergenic than the toxic strain (see Supplemental Material, Figure S1).

**Table 1:** Patient characteristics and rhinitis diagnoses.

Patient	Age (years)	Sex	Race	Atopy	Diagnosis
1	61	M	C	No	NAR
2	58	M	C	No	Asthma
3	45	M	C	Yes	PAR
4	25	F	C	Yes	PAR
5	20	M	C	No	NAR
6	31	F	C	Yes	PAR
7	27	F	B	Yes	PAR, AD, FA
8	26	F	B	Yes	MR
Abbreviations: AD, atopic dermatitis; B, African American; C, Caucasian; F, female; FA, food allergy; M, male; MR, mixed rhinitis; NAR, nonallergic rhinitis; PAR, perennial allergic rhinitis.					

An IgE-specific immunoblot was performed to further characterize the immune-reactive peptides eliciting these responses. Three bands (> 50 kDa) were identified in all patients’ sera (Figure 1B) for both the MC(+) and MC(–) lysates. Although the intensities of the bands varied among individual patient serum and across various bands for the same serum, the overall trend was consistent. No bands were identified using pooled sera from healthy controls. Interestingly, the quantification of immunoblot bands shows that both the MC(–) and the MC(+) strains contain similar amounts of IgE binding peptides (see Supplemental Material, Figure S2). These results indicate that IgE binds to *M. aeruginosa* peptides present in lysates of both strains. Because the immunoblot was performed under denaturing conditions, the results also suggest that the MC(+) lysate may contain an endogenous inhibitor that prevents effective IgE binding to the relevant peptide(s) in its native form (Figure 1A).

**Figure 2:**
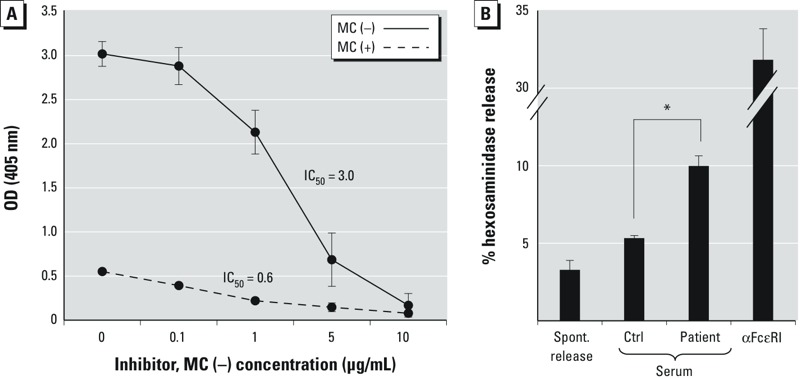
(*A*) ELISA inhibition using pooled serum from SPT-positive patients. Patient serum was preincubated with increasing concentrations of MC(–) lysate (solution phase) before being added to wells coated with MC(–) and MC(+) lysates (solid phase). Results are expressed as optical densities (OD 405 nm) and are representative of four independent experiments. IC_50_ values are indicated. (*B*) Rat basophil leukemia cells (RBL SX‑38) were sensitized with pooled serum from either *M. aeruginosa*–sensitized patients or from controls (Ctrl) before being exposed to MC(–) lysate. Spont., spontaneous. Anti-FcεRI was used as a positive control. Data are representative of two separate experiments. *p < 0.01 compared with pooled control serum, by unpaired Student’s *t*-test.

In vitro *functional characterization of* M. aeruginosa *extracts*. To further demonstrate the specificity of the *M. aeruginosa* peptides identified in the serum of sensitized patients, we performed an ELISA inhibition assay using plates coated with MC(+) and MC(–) lysates. Using the MC(–) lysate as the serum inhibitor, specific IgE binding was reduced in a dose-dependent manner (Figure 2A). The IC_50_ (concentration required for 50% inhibition) values were 0.6 and 3.0 μg/mL for MC(+) and MC(–) lysates, respectively. These data confirm that the same allergen is present in both lysates and suggest that the MC(+) lysate may contain an endogenous inhibitor that prevents IgE from effectively binding to the specific allergen (Figure 2A, 0 μg/mL inhibitor).

We then assessed whether the MC(–) lysate could release mediators using an *in vitro* functional assay. For the functional assay, we used a rat basophil leukemic cell line (RBL SX-38) that expresses a high-affinity human IgE receptor (FcεR1) (Dibbern et al. 2003). Prior to performing the cell-based assay, we evaluated the toxicity of the MC(+) and MC(–) lysate in RBL SX-38 cells. Although the MC(+) lysate showed an obvious dose-dependent toxicity after a 48-hr treatment, the MC(–) lysate had a minimal effect on cell viability (see Supplemental Material, Figure S3). In addition, during the β-hexosaminidase release assay, exposure to the lysate was limited to 30 min, thus reducing any potential toxic effects the *M. aeruginosa* lysates have on the cells. As shown in Figure 2B, pooled serum from patients 3–6 (Figure 1A) released approximately 10% of the total mediator content measured as β-hexosaminidase (a surrogate marker for histamine), which was significantly greater than spontaneous release or a negative control. This result indicates that the MC(–) lysate could induce β-hexosaminidase release and confirms the functional relevance of the *M. aeruginosa*–specific IgE responses identified by SPT and specific IgE ELISA.

**Figure 3:**
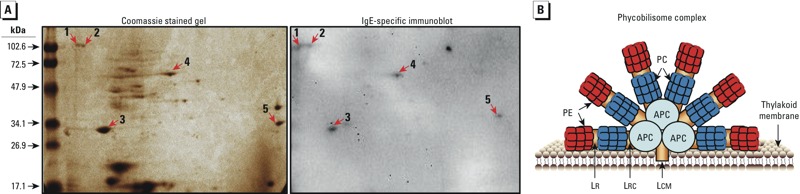
(*A*) 2D gel electrophoresis using MC(–) lysate (left) followed by specific IgE immunoblot (right) using pooled serum from SPT-positive patients demonstrates five immunoreactive spots. These spots were identified by mass spectrometry as phycocyanin (2, 3, and 4) and LCM peptide (1 and 5), which are components of the *M. aeruginosa* phycobilisome complex. (*B*) Schematic diagram depicting the proteins of the phycobilisome complex (modified with permission from Guan et al. 2007). Abbreviations: APC, allophycocyanin; LCM, linker core-membrane; LR, linker rod; LRC, linker rod core; PC, phycocyanin; PE, phycoerythrin.

*Identification of* M. aeruginosa *immunogenic peptides*. To identify the allergen(s) in the cyanobacteria that is binding to IgE, we performed 2D gel electrophoresis, followed by immunoblotting, using MC(–) lysate and pooled patient sera. In the immunoblot, five spots that bind to specific IgE in patient sera were identified (Figure 3A). Mass spectrometry identified two as the linker core-membrane peptide (LCM); the other three spots were identified as the β subunits of phycocyanin of *M. aeruginosa.*

Phycocyanin is composed of two subunits: alpha and beta. The molecular weights (MWs) of the alpha and beta subunits are 18 and 20 kDa, respectively, while that of LCM is 120 kDa. LCM is responsible for anchoring the phycobilisome complex to the thylakoid membrane, which is the structural unit of the grana (stacks of thylakoid membranes) containing the chlorophyll in cyanobacteria (Figure 3B). In its native form, phycocyanin mostly exists as hexamers [(αβ)_6_] held together by intermediate and weak hydrogen bonds (Contreras-Martel et al. 2007). Upon disassembly from the thylakoid membrane (e.g., cell lysis by sonication), the hexamers [(αβ)_6_] rapidly disintegrate into trimers [(αβ)_3_] and monomers (αβ) (Mörschel et al. 1980), which explains the three bands shown in Figure 1B and three of the five spots in Figure 3A. These results indicate that phycobiliproteins—either LCM, phycocyanin, or both—are potentially responsible for the allergenicity observed with *M. aeruginosa.*

**Figure 4:**
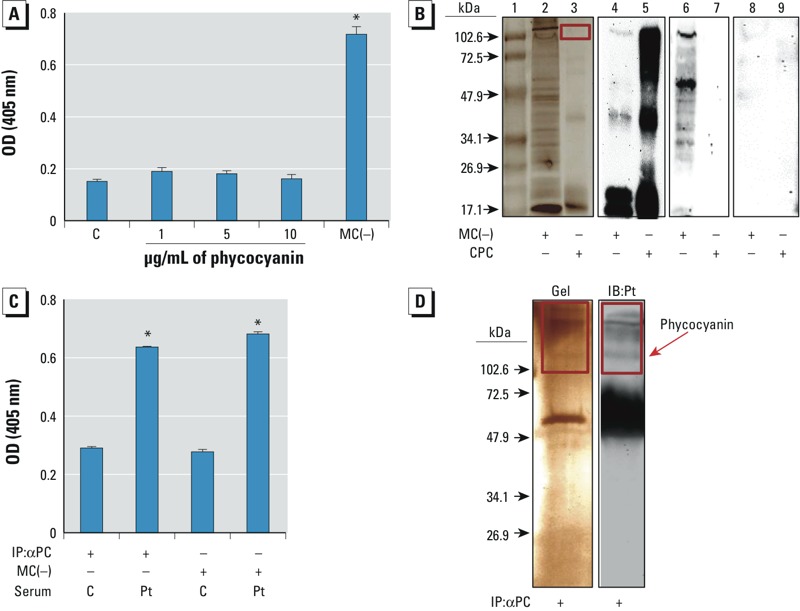
(*A*) IgE-specific ELISA using commercially purified phycocyanin (CPC) incubated with pooled serum from SPT-positive patients or controls (C). Nontoxic strain lysate [MC(–)] incubated with serum from SPT-positive patients was used as positive control. (*B*) IgE-specific gel and immunoblot analysis using CPC or MC(–) lysate incubated with pooled serum from SPT-positive patients or controls. Lanes 1–3 are results of SDS‑PAGE [lane 1, MW markers; lane 2, MC(–) lysate; lane 3, CPC]; lanes 4–9 are results of immunoblots [lanes 4 and 5 are MC(–) lysate and CPC, respectively, labeled with anti-PC antibody; lanes 6 and 7 are MC(–) lysate and CPC, respectively, incubated with pooled serum from *M. aeruginosa*–sensitized patients; lanes 8 and 9 are MC(–) lysate and CPC, respectively, incubated with pooled control sera]. The red rectangles indicate phycocyanin and phycocyanin-binding proteins. (*C*) IgE-specific ELISA using immunoprecipitated phycocyanin (IP:αPC) from MC(–) lysate incubated with pooled serum from SPT-positive patients (Pt) or controls (C). (*D*) Coomassie-stained SDS-PAGE gel (left) and IgE-specific immunoblot of immunoprecipitated PC (IP:αPC; right) from MC(–) lysate incubated with pooled serum from SPT-positive patients. Red rectangles indicate phycocyanin and phycocyanin-associated proteins; the dense band below phycocyanin (~ 50 kD) in the gel and blot represent the IgG heavy chain from αPC antibody. *p < 10^–5^ by unpaired Student’s *t*-test.

*IgE reactivity of purified and endogenous phycocyanin*. Because phycocyanin has previously been reported to cause anaphylaxis (Petrus et al. 2010) and was identified as one of the cyanobacteria allergens (Figure 3A), we performed an IgE-specific immunoblot and ELISA to test the IgE reactivity of commercially purified phycocyanin. Although phycocyanin was detected using anti-phycocyanin antibody (αPC), it did not show any specific IgE reactivity on ELISA (Figure 4A) nor did it reveal any bands on the immunoblot (Figure 4B) using pooled sera from sensitized patients.

Because multiple bands were detected by αPC when the MC(–) lysate was used, we asked whether immunoprecipitated phycocyanin could elicit IgE-specific reactivity. Indeed, endogenous phycocyanin did possess IgE reactivity on the ELISA and immunoblot (Figure 4C,D), indicating that phycocyanin in its native form—either alone or in a complex with other interacting phycobiliproteins (potentially LCM)—is the relevant cyanobacteria allergen.

**Figure 5:**
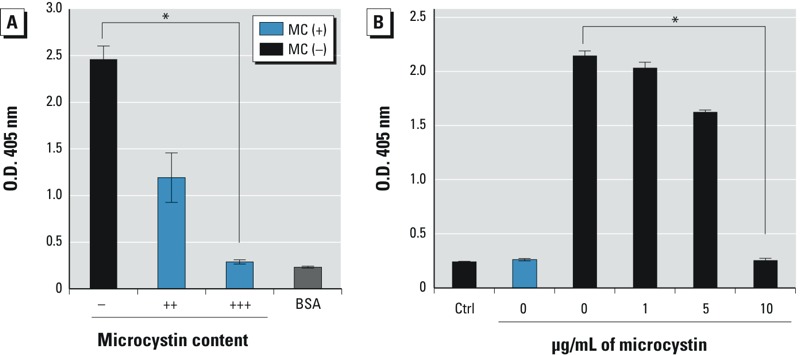
(*A*) IgE-specific reactivity (OD 405 nm). Lysates from different *M. aeruginosa* strains producing varying amounts of microcystin were incubated with pooled sensitized patient sera; BSA was used as a negative control. Abbreviations: –, no microcystin; ++, medium microcystin content (0.1 ng/mL); +++; high microcystin content (241.6 ng/mL); BSA, bovine serum albumin. (*B*) Inhibition of IgE-specific reactivity (OD 405 nm). MC(–) lysates preincubated with extracts containing various concentrations of purified microcystin were incubated with pooled serum from sensitized patients or controls (Ctrl). Data are representative of three separate experiments. *p < 10^–7^, by unpaired Student’s *t*-test, for “+++” compared with “–” in (*A*) and for 10 μg/mL microcystin compared with 0 μg/mL microcystin in (*B*).

*Effect of microcystin on the allergenicity of* M. aeruginosa. The most apparent difference between both *M. aeruginosa* strains is their capability of producing or not producing microcystin toxin. Only the toxic strain contains the gene that codes for microcystin (Ouellette and Wilhelm 2003). Interestingly, although we observed that the nontoxic strain always elicited a strong IgE-specific response consistent with allergenicity, the IgE responses elicited by different batches of the toxic strain varied tremendously. We therefore asked whether microcystin in the toxic strain could be preventing or interfering with it becoming allergenic. To this end, we measured the microcystin content in the lysates of three different batches of *M. aeruginosa* and performed a direct IgE-specific ELISA assay. The nontoxic strain demonstrated strong IgE reactivity (Figure 5A), but a significantly reduced reactivity was observed in the toxic strain lysate with medium (++; 0.1 ng/mL) microcystin content. No reactivity was seen in the lysate with high (+++; 241.6 ng/mL) microcystin content, suggesting that the allergenic activity of the lysate was inversely associated with the microcystin content. To further evaluate the effects of microcystin on the allergenicity of *M. aeruginosa,* an indirect ELISA was performed in which the nontoxic strain lysate was preincubated with increasing concentrations of purified microcystin, ranging from 1 to 10 μg/mL (Figure 5B). At low concentrations, microcystin had very little effect on the lysate, whereas at concentrations ≥ 5 μg/mL, microcystin caused a significant decrease in the ability of the lysate to bind IgE. At 10 μg/mL microcystin, the nontoxic strain lysate resembled the toxic strain lysate, exhibiting almost no IgE reactivity. These results suggest an interaction between microcystin and the relevant cyanobacteria epitope(s) that interferes with its allergenicity.

## Discussion

We found that both phycobiliproteins, LCM and phycocyanin, bind to IgE in sera of chronic rhinitis patients previously found to elicit positive SPT responses to *M. aeruginosa* extracts (Bernstein et al. 2011). The relevance of *M. aeruginosa* extracts containing the phycobilisome complex in eliciting a specific IgE response was confirmed by demonstrating mediator release in a rat basophil cell line expressing the human high-affinity IgE receptor, FcεR1. Interestingly, the ability of *M. aeruginosa* strain lysates to bind specific IgE appears to be inhibited by the presence of microcystin because non-microcystin-producing strains were more allergenic than microcystin-producing strains. Furthermore, when microcystin was added to the lysate from the non-microcystin-producing strain, IgE binding was significantly attenuated, indicating an antagonistic interaction between microcystin and allergen. Although the allergenic potential of phycocyanin has previously been reported in humans (Cohen and Reif 1953; Petrus et al. 2010), this is the first report demonstrating a specific IgE response using human sera from chronic rhinitis patients, with or without IgE-mediated sensitization to common aeroallergens. In addition, this is the first study that demonstrates that other proteins of the phycobilisome complex, specifically LCM, may be important for eliciting IgE-mediated cyanobacteria sensitization.

Purified phycocyanin, because of its brilliant blue color, is often used as a dye in cosmetic products, as a food coloring, and as a fluorescent marker in biomedical research (Domínguez 2013; Glazer 1994; Yoshida et al. 1996). Whereas commercially purified phycocyanin failed to elicit an IgE response in this study (Figure 4A,B), phycocyanin that contained one or more of the phycocyanin-interacting proteins of the phycobilisome complex did elicit such a response (Figure 4C,D). This finding is in agreement with a previous report by Petrus et al. (2010), who identified phycocyanin, extracted from the cyanobacterium *Arthrospira platensis* (spirulina), was responsible for inducing an anaphylactic reaction in a teenage patient who ingested spirulina-containing food supplement tablets. The IgE-binding allergen was found to be in its native form (i.e., homogenized spirulina tablets containing the phycobilisome complex), similar to what we have observed (Petrus et al. 2010). In addition, Cohen and Reif (1953) performed skin-patch testing using phycocyanin extracted from *Anabaena* sp. to confirm delayed-type hypersensitivity in a patient presenting with a contact-dermatitis–appearing skin rash suspected to be secondary to cyanobacteria. Although this represented a delayed-type reaction consistent with T-cell–mediated hypersensitivity, the potential for endogenous phycocyanin to elicit a specific allergic immune-mediated response in a susceptible individual was clearly evident.

An important finding in the present study was the inhibitory effects of microcystin on IgE-mediated allergenicity of *M. aeruginosa* non-toxin–containing strains. Interestingly, this is not the first study to investigate microcystin content and allergenicity. Torokne et al. (2001) performed the maximization sensitization test, an indirect and less sensitive method used to induce delayed type sensitization, in guinea pigs in order to study the allergenic properties of six cyanobacteria strains. Similar to what we have found, the most toxic strain, *M. aeruginosa* (2.21 mg/g microcystin), was nonallergenic, whereas the species with undetectable amounts of microcystin, *Aphanizomenon flos-aquae*, was the most sensitizing (Torokne et al. 2001). However, these investigators failed to identify a correlation between microcystin content and allergenicity and concluded that the observed allergenicity was more likely due to lipopolysaccharides (LPS) from contaminating bacterial flora rather than directly from cyanobacteria because allergenicity was not observed in axenic strains. This is in contrast to our study, which isolated phycobilisome proteins from axenic strains of cyanobacteria and demonstrated specific IgE responses to nontoxic *M. aeruginosa* strains using sera from *M. aeruginosa*–sensitized patients confirmed by SPT, serologic-specific IgE responses, and functional β-hexosaminidase assays. Furthermore, we were able to directly and indirectly demonstrate the inhibitory effect of microcystin on allergenicity (Figure 5B). A major difference between the Torokne et. al. (2001) study and our findings is that they compared microcystin effects across various strains of cyanobacteria, not taking into account whether or not microcystin was the major toxin for that species, whereas we specifically investigated microcystin, which is the major toxin in *M. aeruginosa.* Because other toxins in these species, which were not measured, could have a similar effect on allergenicity that we observed with microcystin in *M. aeruginosa*, Torokne et. al. (2001) could have missed the significant inverse correlation between toxicity and allergenicity. Other differences between the findings of Torokne et. al. (2001) and the present study can be explained by the differential responses observed with animal models versus *in vitro* experiments using human serum, as well as differences between the methodologies used to purify cyanobacteria proteins and to elicit sensitization. It should be mentioned that three patients in our study (Figure 1; patients 3, 5, and 8) also exhibited a positive SPT to *A. flos-aquae* extracts (Bernstein et al. 2011), suggesting a potential cross reactivity between the allergen(s) in *M. aeruginosa* and *A. flos-aquae.* This may indicate that the specific IgE reactivity observed could be generalized to all cyanobacteria species, specifically non-microcystin-producing species, although such a generalization would require further investigation.

The interaction between microcystin and phycocyanin complex, leading to the suppression of IgE binding to the phycocyanin complex, is unclear. However, the facts that both phycocyanin and LCM can be phosphorylated (Mann and Newman 1999; Piven et al. 2005) and that microcystin is a phosphatase inhibitor (MacKintosh et al. 1995; Sim and Mudge 1993) suggest a mechanistic basis for a possible interaction. Furthermore, a number of studies (Jüttner and Lüthi 2008; Vela et al. 2008; Zilliges et al. 2011) have reported the binding of microcystin to phycobiliproteins, particularly microcystin binding to phycocyanin and allophycocyanin (Jüttner and Lüthi 2008). Also, because 78% of microcystin in *M. aeruginosa* cells colocalizes with the thykaloid membrane (Young et al. 2005) and is, therefore, in close proximity to the phycobilisome complex, it is possible that microcystin directly or indirectly interferes with the IgE binding epitope of this complex. Similar mechanisms have been previously proposed for targeting novel therapies to inhibit mast cell activation (Shenker et al. 2010). To further complicate matters, several investigators have reported immunomodulatory effects of microcystin using a variety of immune system cell types, even at nontoxic concentrations (Chen et al. 2004, 2005; Hernández et al. 2000; Lankoff et al. 2004; Yea et al. 2001). For example, in mice, microcystin showed a concentration-dependent inhibition of LPS-induced antibody production when both *in vitro* and *in vivo* antibody-forming cell assays were used (Yea et al. 2001). Therefore, further studies are required to better understand the exact mechanism and dynamics by which microcystin inhibits IgE binding to allergen(s) in the phycobilisome complex.

The most important limitation of the present study is that we were unable to isolate and test the allergenicity of LCM and phycocyanin separately or in combination. This proved to be difficult using techniques based on MW and isoelectric point because of their very similar MWs (*M. aeruginosa* approximately 120 kDa) and virtually identical isoelectric points (Figure 3A). In addition, immunochemical studies have previously shown that phycocyanin and LCM from all cyanobacteria sources are closely related in terms of their antigenic and immunogenic potential (Berns 1967; Zilinskas and Howell 1986). Thus, although the phycocyanin-immunoprecipitated complex that we isolated is allergenic, it is difficult to make a definitive conclusion regarding the exact source of antigenic epitope.

The source and duration of exposure to *M. aeruginosa* causing sensitization of the patients previously studied by our group is unknown. It is possible that direct cyanobacteria contact or exposure to consumer products containing phycocyanin as an additive is a contributing factor. Our findings may have important implications for public health because many people are continuously being exposed to cyanobacteria through a variety of settings, including outdoor lakes and pools and indoor aquariums, and potentially through exposure to phycocyanin-containing cosmetic and food coloring products. Two of the patients who exhibited positive SPT and specific IgE responses to cyanobacteria were diagnosed as being nonallergic because they were not sensitized to other aeroallergens (Figure 1). This is interesting because it confirms that there are allergens similar to cyanobacteria that can cause sensitization in patients who would otherwise be diagnosed as nonallergic based on negative skin testing to conventional indoor and outdoor allergens.

## Conclusion

To our knowledge, this is the first study to demonstrate that non-toxin–producing strains of cyanobacteria are more allergenic than toxin-producing strains in chronic rhinitis patients sensitized to cyanobacteria and that the toxin content of the organism has an inhibitory effect on allergenicity. These findings have broad implications for the relevance of ongoing water management strategies to control cyanobacteria blooms, and the findings emphasize the need for further mechanistic and clinical investigation to better understand the health impact of cyanobacteria exposure on susceptible subsets of the population.

## Supplemental Material

(200 KB) PDF
