# Supplementary material for: Identification of Microcystis aeruginosa Peptides Responsible for Allergic Sensitization and Characterization of Functional Interactions between Cyanobacterial Toxins and Immunogenic Peptides
Source: Environ Health Perspect. 2015 Apr 22;123(11):1159–66. doi: 10.1289/ehp.1409065 (PMC4629744; doi:10.1289/ehp.1409065)
Supplement: (200 KB) PDF [file ehp.1409065.s001.acco.pdf]

Environ Health Perspect
doi: 10.1289/ehp.1409065

Note to Readers: *EHP* strives to ensure that all journal content is accessible to all readers. However, some figures and Supplemental Material published in *EHP* articles may not conform to 508 standards due to the complexity of the information being presented. If you need assistance accessing journal content, please contact ehp508@niehs.nih.gov. Our staff will work with you to assess and meet your accessibility needs within 3 working days.

Supplemental Material

Identification of *Microcystis aeruginosa* Peptides Responsible for Allergic Sensitization and Characterization of Functional Interactions between Cyanobacterial Toxins and Immunogenic Peptides

Esmond N. Geh, Debajyoti Ghosh, Melanie McKell, Armah A. de la Cruz, Gerard Stelma, and Jonathan A. Bernstein

Table of Contents

Figure S1. IgE-specific ELISA with different batch lysates (A and B) of *M. aeruginosa* crude cell extracts from toxic, MC(+) and nontoxic, MC(-) strains using individual patient serum from *M. aeruginosa* SPT-positive patients (1-8) and a non-atopic control (C). A paired student's t-test was performed and the asterisks indicate a statistical significance difference ($p<0.01$) between MC(+) and MC(-) strains.

Figure S2. Specific IgE Western blot quantification. The intensity of individual bands from each lane of the western blot image (Figure 1B) was quantified using Labworks software (Ultra-Violet Products Ltd, Upland, CA). The total intensity represents the sum total of individual IgE binding proteins within each lane.

Figure S3. Cytotoxicity Assay. Rat basophil leukemia cells (RBL SX-38) were seeded at 10^4 cells per well in a 96-well plate. At 90% confluence, the cells were either left untreated or treated for 48 hours with varying concentrations of M. aeruginosa toxic strain [MC(+)] and nontoxic

strain [MC(-)] lysates. At the end of the treatment, CytoScan-WST-1 cell toxicity kit (G-bioscience, St. Louis, MO) was used to measure the cytotoxic effect of the lysates per manufacturer's protocol. Percent cytotoxicity was calculated as follows: % Cytotoxicity = (100 x (Cell Control–Experimental)) ÷ (Cell Control). Asterisk ($p<0.05$) and triple asterisks ($p<10^{-5}$) indicate a significance difference from untreated cells using an unpaired Student's t-test.

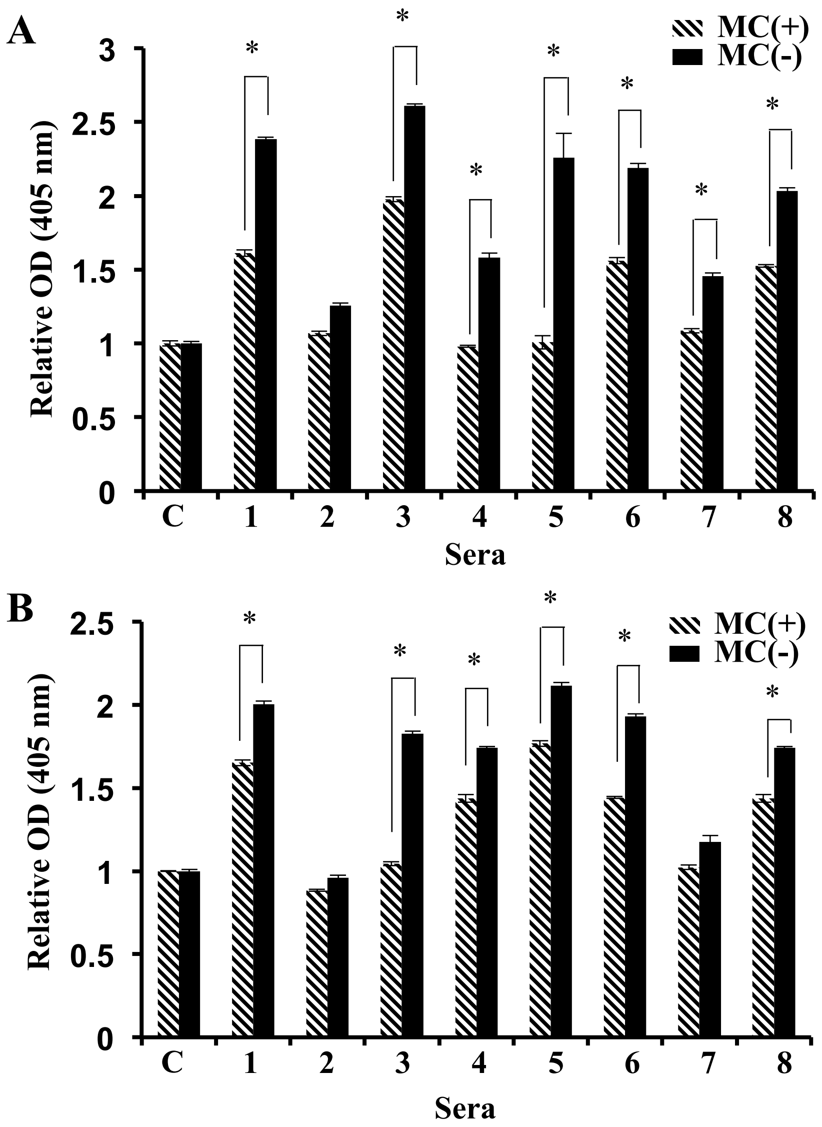

Figure S1. IgE-specific ELISA with different batch lysates (A and B) of *M. aeruginosa* crude cell extracts from toxic, MC(+) and nontoxic, MC(-) strains using individual patient serum from *M. aeruginosa* SPT-positive patients (1-8) and a non-atopic control (C). A paired student's t-test was performed and the asterisks indicate a statistical significance difference ($p<0.01$) between MC(+) and MC(-) strains.

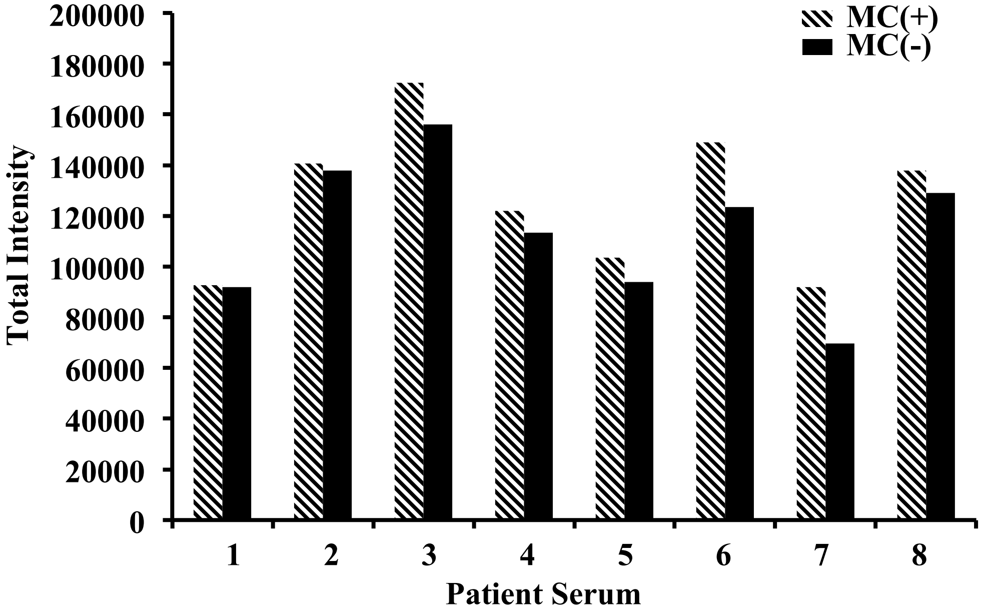

Figure S2. Specific IgE Western blot quantification. The intensity of individual bands from each lane of the western blot image (Figure 1B) was quantified using Labworks software (Ultra-Violet Products Ltd, Upland, CA). The total intensity represents the sum total of individual IgE binding proteins within each lane.

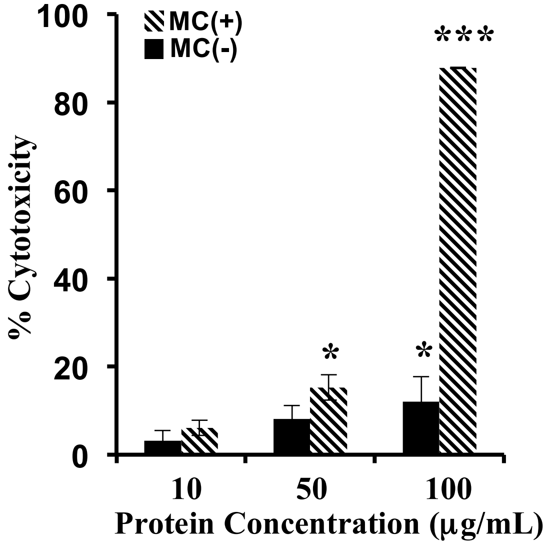

Figure S3. Cytotoxicity Assay. Rat basophil leukemia cells (RBL SX-38) were seeded at 10^4 cells per well in a 96-well plate. At 90% confluence, the cells were either left untreated or treated for 48 hours with varying concentrations of M. aeruginosa toxic strain [MC(+)] and nontoxic strain [MC(-)] lysates. At the end of the treatment, CytoScan-WST-1 cell toxicity kit (G-bioscience, St. Louis, MO) was used to measure the cytotoxic effect of the lysates per manufacturer's protocol. Percent cytotoxicity was calculated as follows: % Cytotoxicity = (100 x (Cell Control–Experimental)) ÷ (Cell Control). Asterisk ($p<0.05$) and triple asterisks ($p<10^{-5}$) indicate a significance difference from untreated cells using an unpaired Student's t-test.
